# Reduced expression levels of let-7c in human breast cancer patients

**DOI:** 10.3892/ol.2015.2877

**Published:** 2015-01-14

**Authors:** XIN-XIN LI, SHU-YAN GAO, PING-YU WANG, XUE ZHOU, YOU-JIE LI, YUAN YU, YUN-FEI YAN, HAN-HAN ZHANG, CHANG-JUN LV, HUI-HUI ZHOU, SHU-YANG XIE

**Affiliations:** 1 Key Laboratory of Tumor Molecular Biology, Department of Biochemistry and Molecular Biology, Binzhou Medical University, Yantai, Shandong 264003, P.R. China; 2 Department of Clinical Medicine, Affiliated Hospital of Binzhou Medical University, Yantai, Shandong 264003, P.R. China; 3 Department of Pathology, Affiliated Yuhuangding Hospital, Medical College of Qingdao University, Yantai, Shandong 264000, P.R. China

**Keywords:** circulating miRNA, breast cancer, let-7c, predictive factor, receiver operating characteristic analysis

## Abstract

Circulating microRNAs (miRNAs) are important in the diagnosis of a number of diseases, since serum or plasma miRNAs are more stable compared with miRNA isolated from blood samples. The aim of the present study was to investigate the association between the expression levels of serum let-7c miRNA and the clinical diagnosis of breast cancer (BC). The circulating let-7c levels of 90 BC patients and 64 healthy controls were determined by performing a reverse transcription-quantitative polymerase chain reaction assay. The results demonstrated that let-7c expression was downregulated in the BC tissues compared with the paracarcinoma control tissues. In addition, the let-7c expression in the serum of BC patients was significantly lower compared with the healthy controls (P<0.01). Using a cutoff value of 0.374×10^3^ copies/ml, the serum expression levels of let-7c exhibited 87.5% sensitivity and 78.9% specificity for distinguishing BC patients from healthy controls (area under the receiver operating characteristic curve, 0.848; 95% confidence interval, 0.785–0.911). Furthermore, the results demonstrated that the serum expression levels of let-7c were significantly higher in premenopausal compared with postmenopausal patients (P<0.05), supporting the hypothesis that postmenopausal status may affect the serum expression levels of let-7c. However, no statistically significant differences were detected in the serum levels of let-7c between ER (or PR)-positive and -negative patients. Therefore, the current study hypothesized that serum let-7c may be used as a novel and valuable biomarker for the diagnosis of BC.

## Introduction

MicroRNAs (miRNAs), a class of small and non-coding RNAs (length, 19–25 nucleotides), have the potential to regulate ~30% of human genes, which may attribute to the development of cancer (1–3). miRNAs regulate gene expression by binding to the 3′-untranslated region (3′-UTR) of the targeted mRNAs. Numerous studies have indicated that abnormal expression of miRNA is associated with the development and progression of various types of human cancer (4–6). Although the biological function of miRNAs remains unknown, specific miRNAs have functions similar to tumor suppressors or oncogenes. Previous studies investigating the expression levels of miRNAs in cancer have indicated their significance and potential use in the classification of cancer or as diagnostic indicators (5,7).

Breast cancer (BC) is one of the most common types of carcinoma and results in a high female mortality rate worldwide (1). In China, the number of BC-associated mortality rate has increased markedly in recent years (3,7). Although early detection of BC is important in order to reduce the mortality rate of this disease, the methods available for primary detection, including mammography, ultrasonography and magnetic resonance imaging, may result in misdiagnosis or missed diagnosis. The expression pattern of each miRNA molecule varies in different cancer phenotypes (including BC) and, thus, can be used in tumor classification, diagnosis, therapy and prognosis (4). Over the past decades, the association between miRNAs and human BC has been extensively investigated (8–10). Previous studies have demonstrated that the expressional changes of a number of miRNAs are involved in BC development and progression (2,5), while serum or plasma miRNA expression levels were found to be different in BC patients and healthy controls (3). Therefore, miRNAs represent a novel approach to BC diagnosis.

The present study aimed to further investigate the association between serum miRNA expression levels and the clinical diagnosis of BC. The circulating let-7c levels were determined in the serum of BC patients and healthy controls by performing individual-based reverse transcription-quantitative polymerase chain reaction (RT-qPCR) assays, in accordance with previous reports (2,3,11). In addition, the present study investigated whether the serum let-7c level can be used as a potential biomarker for BC diagnosis.

## Materials and methods

### Study subjects

The present study was conducted in the Inpatient Department of Medical Oncology at the Affiliated Hospital of Binzhou Medical University (Yantai, China) and performed in accordance with the relevant guidelines of the Medical Ethics Committee of Binzhou Medical University.

In total, 90 females with BC (age range, 27–79 years), who were pathologically diagnosed with BC for the first time between June 1st 2010 and July 31st 2013, were included in the present study. The patients had not been previously treated with chemotherapy or postmenopausal hormone therapy. In addition, 64 healthy controls were recruited from individuals who visited the Affiliated Hospital of Binzhou Medical University for physical examination within the same time period and were not diagnosed with a tumor or physical illness. Prior to enrollment in the present study, written informed consent was obtained from each individual.

### Immunohistochemistry

Fresh BC and healthy tissues from patients who underwent surgery at the Affiliated Hospital of Binzhou Medical University were obtained at the time of surgery, and immediately prepared for pathological diagnosis, immunohistochemistry or RNA isolation. The cancer sample sections containing a minimum of 60% cancer cells were used in the experiments. All patients provided their written consent to participate in this study. Subsequently, the tissues were fixed in 4% paraformaldehyde, embedded in paraffin and cut into 5-μM sections. Subsequently, the sections were deparaffinized in xylene, rehydrated and antigen retrieval was performed by incubation in 10 mM citrate buffer, pH 6.0 at 95–100°C for 10 min. This was followed by washing in phosphate-buffered saline (PBS). To quench the endogenous peroxidase activity, the slides were incubated in 3% hydrogen peroxide for 15 min at room temperature. Next, the BC slides were incubated in 10% normal goat serum [Beijing Zhongshan Golden Bridge Biotechnology Co., Ltd., Beijing, China] for 20 min at room temperature to prevent non-specific staining. Subsequently, the slides were incubated at 4°C overnight with appropriate dilutions of the following primary antibodies: Rabbit monoclonal anti-estrogen receptor (ER; 1:200); and rabbit monoclonal anti-progesterone receptor (PR; 1:200; Beijing Zhongshan Golden Bridge Biotechnology Co., Ltd.). Next, the samples were incubated with horseradish peroxidase-conjugated goat anti-rabbit secondary antibodies (1:5000, Beijing Zhongshan Golden Bridge Biotechnology Co., Ltd.). The negative controls were incubated in 1× PBS without antibody, following the same procedure. The samples was visualized using an ABC kit (Beijing Zhongshan Golden Bridge Biotechnology Co., Ltd.) and positive ER and PR status was considered when nuclear staining was >10%. The expression of ER and PR was examined under the Olympus BX51 AX-70 microscope (Olympus, Tokyo, Japan).

### Sample collection

Whole blood samples (3 ml) were obtained from each subject. The serum samples were separated by centrifugation at 2,650 × g for 10 min at room temperature and stored at −80°C prior to analysis (11).

### miRNA isolation from serum or tissue samples

miRNA was extracted from the serum samples using the mirVana™ miRNA isolation kit (Ambion Life Technologies, Carlsbad, CA, USA) according to the manufacturer’s instructions. The BC tissues were homogenized in a denaturing lysis solution and treated with TRIzol reagent (Invitrogen Life Technologies, Carlsbad, CA, USA) to extract total RNA, according to the manufacturer’s instructions. Subsequently, the mirVana™ miRNA isolation kit was used to obtain the miRNAs from 30–50 mg total RNA samples.

### RT-qPCR

Poly(A) tails were added to the extracted miRNAs using poly(A) polymerase (Ambion Life Technologies) and the complementary (c)DNA molecules were synthesized using the 5′-AACATGTACAGTCCATGGATGd(T)30N(A, G, C or T)-3′ primer. RT-qPCR of Let-7c was performed using the following primers: Forward, 5′-GGTTGAGGTAGTAGGTTGTATGGT-3′; and reverse, 5′-AACATGTACAGTCCATGGATG-3′. Each RT-qPCR reaction mixture contained 0.5 μl cDNA, 7.5 μl sterile water, 1 μl forward primer, 1 μl reverse primer and 10 μl of SYBR^®^ Premix Ex Taq™ (Takara Biotechnology Co., Ltd., Dalian, China) and was performed using the Rotor-Gene 3000 system (Corbett Life Science, Mortlake, Australia) as follows: Initial denaturation at 95°C for 5 min, followed by 40 cycles of denaturation at 95°C for 20 sec, annealing at 60°C for 20 sec and extension at 72°C for 30 sec. As described in previous studies, 5S ribosomal RNA was used as the reference control (12,13). All the experiments were performed in triplicate.

### Statistical analysis

The experimental data were initially analyzed for normal distribution and variance homogeneity using the Shapiro-Wilk test and F-test, respectively. Normal distribution data are represented as the mean ± standard deviation, and all the other data are represented as median and quartiles. Differences in the age, height or weight between the BC patients and healthy controls were analyzed using the Student’s t-test. However, when the serum expression levels of let-7c did not demonstrate normal distribution, nonparametric tests were applied to analyze these differences. In addition, continuous variables between the BC patients and controls were analyzed by performing the Wilcoxon rank-sum test. Statistical analyses were performed using R^©^ software (version 2.15.0; http://www.r-project.org). P<0.05 was considered to indicate a statistically significant difference. Furthermore, receiver operating characteristic (ROC) curves were generated using the SPSS software (version 18.0; SPSS, Inc., Chicago, IL, USA) to assess the diagnostic accuracy of each parameter. The area under the ROC curve (AUC) was used to identify the optimal sensitivity and specificity levels at which cancer patients can be distinguished from healthy individuals.

## Results

### Clinical characteristics

The demographic and clinical characteristics of the 90 BC patients and 64 healthy controls that participated in the present study are listed in Table I. No statistically significant differences in the age, height or weight were identified between the BC patients and healthy controls. In addition, no clear statistically significant differences in the menopausal status were identified between the BC patients and healthy controls.

**Figure 1:**
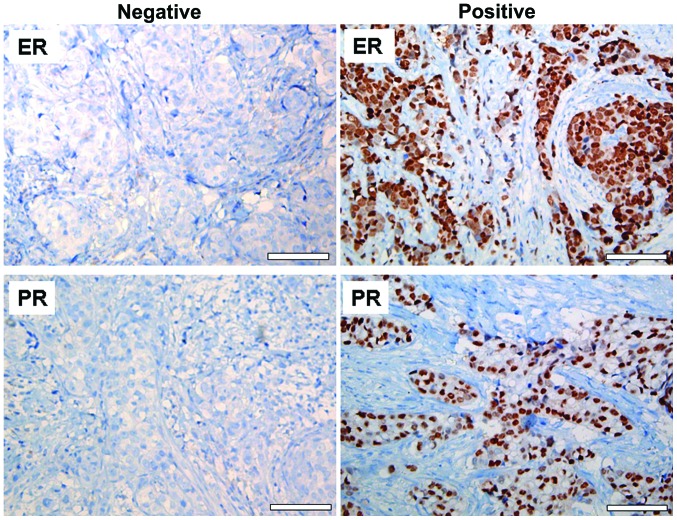
Immunohistochemical analysis of estrogen receptor (ER) and progesterone receptor (PR) expression levels in breast cancer tissues. Using a limit of >10% nuclear staining to define positive ER and PR status, 64 patients were determine as ER-positive and 60 patients as PR-positive. Scale bar, 150 μm.

The presence of ER and PR in the BC tissue samples was detected by immunohistochemical analysis. Nuclear staining of >10% was considered to indicate positivity ER and PR status (Fig. 1). Of the 90 BC patients, 64 patients were ER-positive (71.1%) and 60 were PR-positive (66.6%).

### Reduced let-7c expression levels in BC tissues

**Figure 2:**
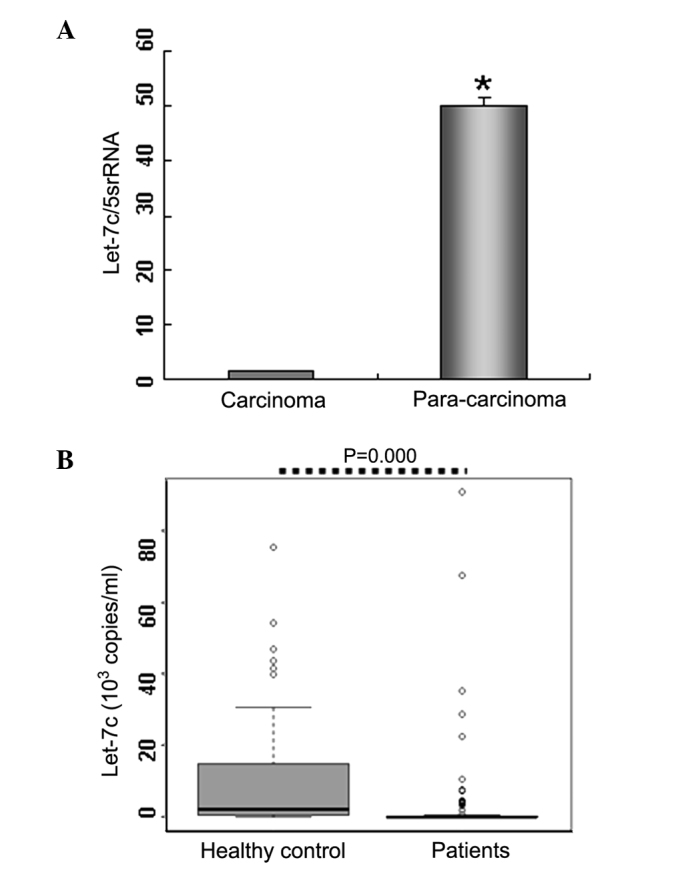
Detection of let-7c expression levels in (A) tissue samples, using reverse transcription-quantitative polymerase chain reaction, and (B) serum samples from breast cancer (BC) patients and heathy controls. let-7c expression was significantly lower in the BC tissues compared with the paracarcinoma tissues (n=4; ^*^P<0.01 vs. carcinoma; control, 5S ribosomal RNA). In addition, the serum let-7c expression levels were lower in BC patients (n=90) compared with the healthy controls (n=64; P=0.000). Data are expressed as the mean ± standard deviation.

Considerable evidence accumulated from a number of previous studies has indicated that downregulation of let-7 family miRNAs may be associated with a poor clinical outcome in BC patients (14,15). Therefore, to investigate the role of let-7c in BC, the present study analyzed the expression levels of let-7c in BC tissues. The results indicated that let-7c expression was markedly decreased (>20-fold lower) in BC tissues (n=4) compared with paracarcinoma tissues (n=4; Fig. 2A), supporting the suppressive role of let-7c in tumor proliferation.

### Reduced serum let-7c expression levels in BC patients

The serum expression levels of let-7c were detected by performing qPCR analysis to investigate the potential role of let-7c in the diagnosis of BC. The results of the present study demonstrated that serum let-7c levels in BC patients (0.035×10^3^ copies/ml; n=90) were significantly lower compared with the healthy controls (2.300×10^3^ copies/ml; n=64; P<0.01; Table I; Fig. 2B). These results were consistent with the let-7c expression levels identified in the BC tissues samples, indicating that let-7c may be an important factor in BC diagnosis.

### Correlation between ER/PR status and circulating let-7c expression levels

**Figure 3:**
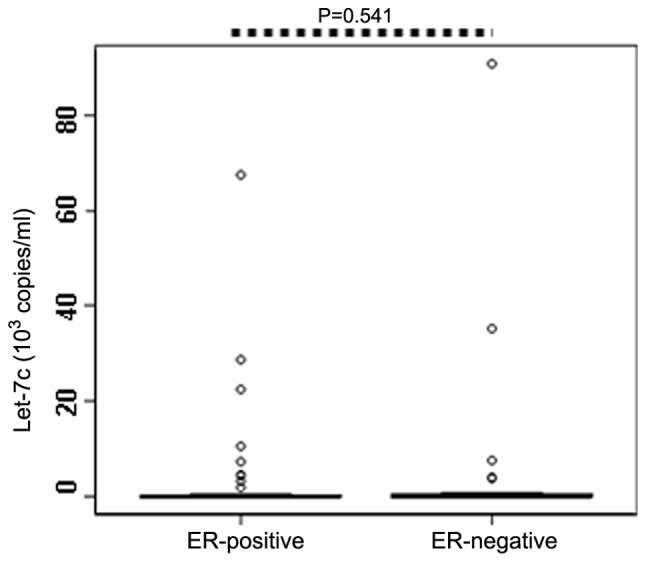
Correlation between serum let-7c expression levels and ER-positive expression in breast cancer (BC) patients. The serum let-7c levels in ER-positive BC patients (n=64) were not significantly different when compared with the ER-negative BC patients (n=26; P=0.541). ER, estrogen receptor; PR, progesterone receptor.

**Figure 4:**
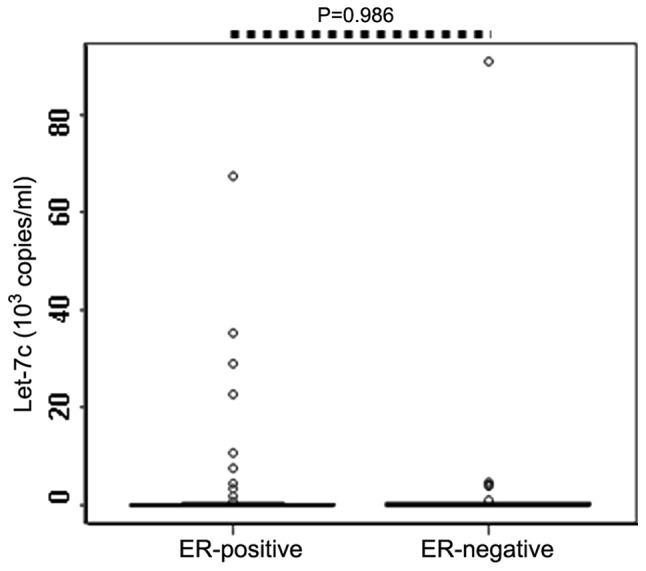
Correlation between serum let-7c expression levels and PR-positive expression in breast cancer (BC) patients. No significant difference was identified between the serum let-7c expression levels of the PR-positive (n=60) and PR-negative BC patients (n=30; P=0.986). PR, progesterone receptor; ER, estrogen receptor.

ER and PR have been previously reported as important factors associated with the etiology and therapeutic treatment strategy of BC (16,17). Therefore, to investigate the correlation between ER or PR status and the serum expression levels of let-7c, let-7c expression was compared between ER- (or PR-) positive and negative patients. The results demonstrated that serum expression levels of let-7c in the ER-positive patients (n=64; 0.033×10^3^ copies/ml) were not significantly different compared with the ER-negative patients (n=26; 0.036×10^3^ copies/ml; P=0.541; Table II; Fig. 3). Similarly, no statistically significant difference was identified between the serum levels of let-7c in the PR-positive (n=60) and PR-negative patients (n=30; P=0.986; Table II; Fig. 4).

### Correlation between menopausal status and circulating let-7c expression levels

**Figure 5:**
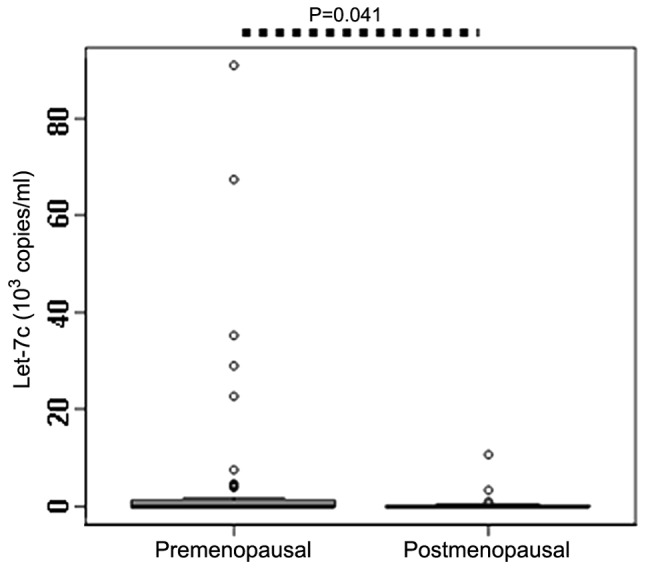
Correlation between serum let-7c levels and menopausal status of breast cancer patients. The serum let-7c levels in premenopausal status patients (n=48) were evidently higher compared with those in postmenopausal status patients (n=42; P=0.040).

Since postmenopausal females with a high breast density exhibit increased risk of developing BC (18), the serum let-7c expression levels were compared between premenopausal and postmenopausal patients. The results identified that the expression levels of let-7c in the premenopausal patients (0.036×10^3^ copies/ml) was significantly higher compared with the postmenopausal patients (0.032×10^3^ copies/ml; P=0.040; Table II; Fig. 5), which indicates that the postmenopausal status may affect the expression level of serum let-7c.

### Diagnostic potential of let-7c expression levels in BC

**Figure 6:**
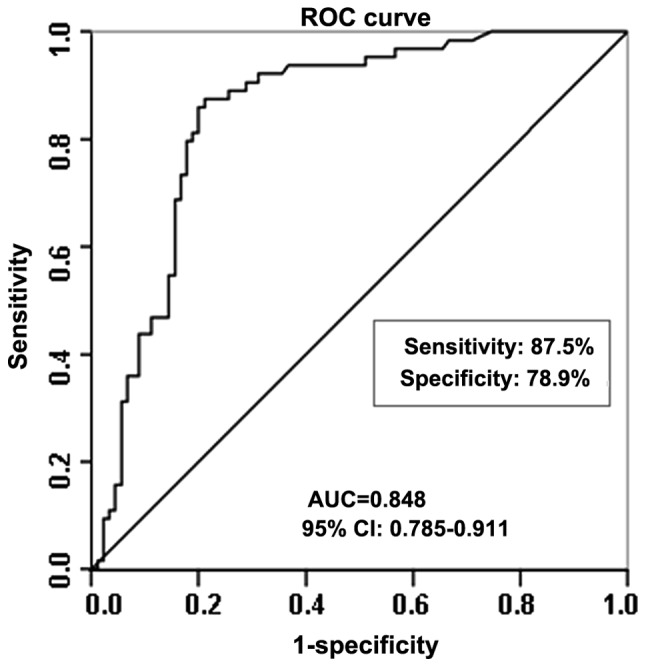
ROC curves for serum let-7c expression levels in breast cancer (BC) patients, indicating an AUC with the diagnostic power to distinguish BC patients from healthy controls. ROC, receiver operating characteristic; AUC, area under the ROC curve; CI, confidence interval.

Statistical ROC analysis was used to investigate the diagnostic potential of let-7c serum expression levels in BC patients. The expression data for let-7c were plotted using the ROC curves to identify a cut-off value that could distinguish breast cancer patients from healthy controls. Using a cutoff level of 0.374×10^3^ copies/ml, the serum expression levels of let-7c presented sensitivity of 87.5% and specificity of 78.9% in distinguishing the BC patients from the healthy controls, with an AUC of 0.848 (95% confidence interval, 0.785–0.911; P<0.001; Fig. 6).

## Discussion

let-7 miRNAs are members of a highly conserved miRNA family consisting of 12 genes (including let-7-a1, -a2, -a3, -b, -c, -d, -e, -f1, -f2, -g, -i and miR-98), which are located on eight different chromosomes (19). Let-7 was initially described in *Caenorhabditis elegans* and functionally conserved from lower invertebrates to higher mammals (20). In the present study, a comprehensive investigation of serum let-7c miRNA expression was conducted in BC patients and healthy controls using RT-qPCR (21). The expression levels of let-7c were significantly decreased in the serum of the BC patients compared with the healthy controls, which indicates that serum let-7c may have a considerable diagnostic function in differentiating BC patients from healthy controls.

The let-7 family members function as tumor suppressors and have been associated with various target genes, including Ras (20), high mobility group AT-hook 2 (22,23) and B-cell lymphoma-extra large (Bcl-xL) (24). let-7 expression is downregulated in a number of malignancies. For instance, let-7 was identified to be decreased in human hepatoma cells and tissues, which are associated with enhanced expression of Bcl-xL (24). A previous study revealed that let-7c was a downregulated epithelial miRNA and its functions were delineated in unique cells derived from columnar cell hyperplasia (25). Similarly, the present study identified that let-7c expression was downregulated in BC tissues compared with paracarcinoma tissues. Thus, the present and aforementioned studies indicated the suppressive role of let-7 miRNAs in tumorigenesis.

A greater number of studies focusing on the function of miRNAs have been conducted, particularly those investigating the roles of circulating miRNAs in disease diagnosis (20). Circulating miRNAs isolated from the serum or plasma are more stable compared with those isolated from the blood (10). In addition, circulating miRNAs are stable at room temperature and can survive under the effects of RNase and DNase (1,11). Thus, the expression patterns of circulating miRNAs may be used as potential diagnostic indicators for a number of diseases, including tumors, improving cancer diagnosis. To further investigate the role of let-7c expression levels in the diagnosis of BC, the circulating let-7c levels were compared between BC patients and healthy controls. It was identified that let-7c expression was lower in the serum of BC patients compared with the healthy controls. Furthermore, at the optimal cut-off, the serum level of let-7c exhibited sensitivity of 87.5% and specificity of 78.9% for distinguishing BC patients from healthy controls.

The important role of pathological analysis in the diagnosis of BC is biomarker testing, specifically the accurate assessment of the ER and PR status of BC tissues (26,27). Previously, significant associations have been reported between tumor size (or the presence of distant metastases) in BC and ER/PR-positive rate (or menopausal status) (28). Therefore, the association between the expression level of let-7c and the ER/PR-positive rate, as well as the menopausal status of the patients, was investigated in the present study. The results indicated that 71.1% of primary BC patients expressed ER, while 66.6% expressed PR. Subsequent investigation into the association between ER (or PR)-positive expression and serum expression levels of let-7c revealed that ER (or PR)-positive expression did not affect the serum expression levels of let-7c. However, let-7c expression in the premenopausal patients was significantly higher compared with the postmenopausal patients, indicating that the postmenopausal status may affect let-7c expression levels. Moreover, Kerlikowske *et al* reported that postmenopausal females with a high breast density presented an increased risk of developing BC (18).

In conclusion, the present study identified that let-7c expression was lower in BC tissues compared with paracancerous tissues. Furthermore, the let-7c serum levels of the BC patients were significantly lower compared with the levels of the healthy controls and were affected by the menopausal status of the patients. Although the results of the current study indicate that serum let-7c expression levels may represent a novel diagnostic biomarker for BC patients, well-designed studies with larger sample sizes are required to further confirm the role of let-7c in cancer diagnosis.

## Figures and Tables

**Table I:** Demographic and clinical characteristics of the study samples.

Parameter	Healthy controls (n=64)	Patients (n=90)	P-value^a^
Age, years^b^	43.781±15.831	47.900±9.882	0.068
Weight, kg^b^	62.638±8.844	65.408±8.238	0.090
Height, cm^b^	160.500±6.414	158.551±4.619	0.075
Estrogen receptor-positive/negative, n	-	64/26	-
Progesterone receptor-positive/negative, n	-	60/30	-
Premenopausal/postmenopausal, n	35/29	48/42	0.998
Median let-7c, ×10^3^ copies/ml	2.300	0.035	<0.01

a P-values were determined by performing a Student’s t test or a Wilcoxon rank-sum test to compare the patient samples with the control samples.

b Values represented as the mean ± standard deviation.

**Table II:** Association of ER, PR and menopausal status with let-7c.

Parameter	Median let-7c, ×10^3^ copies/ml	P-value^a^
ER		0.541
Positive (n=64)	0.033	
Negative (n=26)	0.036	
PR		0.986
Positive (n=60)	0.035	
Negative (n=30)	0.033	
Menopausal status		0.040^a^
Premenopausal (n=48)	0.036	
Postmenopausal (n=42)	0.032	

a P-values were determined using the Wilcoxon rank-sum test to compare premenopausal samples with postmenopausal samples.

ER, estrogen receptor; PR, progesterone receptor.

## Abbreviations

miRNA: microRNA
RT-qPCR: reverse transcription-quantitative polymerase chain reaction
3′-UTR: 3′-untranslated region
BC: breast cancer
CI: confidence interval
ER: estrogen receptor
PR: progesterone receptor
ROC: receiver operating characteristic
AUC: area under the ROC curve
